# Authors’ Response to Peer Reviews of “Administration Technique of Intranasal Corticosteroid Sprays Among Nepali Pharmacists: Cross-Sectional Study”

**DOI:** 10.2196/91445

**Published:** 2026-01-29

**Authors:** Amar Prashad Chaudhary, Suraj Kumar Thakur, Shiv Kumar Sah

**Affiliations:** 1 Tribhuvan University Teaching Hospital, MaharajgunjKathmandu, 0977, Nepal, 977 9860329145; 2 Institute of Medicine, Maharajgunj Medical Campus, Tribhuvan University, Kathmandu, Nepal

**Keywords:** intranasal corticosteroid spray, allergic rhinitis, device use technique, pharmacist, patient counselling, continuing pharmacy education

*This is the authors’ response to peer-review reports for “Administration Technique of Intranasal Corticosteroid Sprays Among Nepali Pharmacists: Cross-Sectional Study.”*

## Round 1 Review

### Reviewer AH [1]

#### General Comments

*This paper [* 2 *] addresses an important gap by evaluating pharmacists’ proficiency in demonstrating intranasal corticosteroid technique, using a standardized 12-step checklist with 5 critical steps. The sample size (n=365) is reasonable for a local study, and the use of multivariate logistic regression and Chi-square automatic interaction detection decision tree analysis adds analytical depth. The findings highlight systemic issues, such as inadequate training and curriculum gaps, which could inform policy changes to improve allergic rhinitis management and reduce adverse effects like epistaxis.*

#### Specific Comments

##### Major Comments

*1. Simple random sampling was used for pharmacies, but details on how wards were selected or how pharmacists within pharmacies were approached are vague. Please supplement and elaborate on further details of the randomization. More information on such would help lower the selection bias (eg, busier or more accessible pharmacies might be overrepresented).*

**Response:** Thank you for this valuable comment. We agree that additional clarification regarding the sampling process is important to demonstrate methodological rigor and minimize concerns about selection bias. In the revised manuscript, we have now expanded the description of the sampling procedure. We have clarified how the wards in Kathmandu district were included, how the list of pharmacies was prepared, how simple random sampling of pharmacies was actually conducted, how pharmacists within each selected pharmacy were approached, and how the accessibility or busyness of pharmacies was handled.

*2. The questionnaire’s validity is only face-validated by experts, with no content or construct validity testing mentioned. Reliability was assessed via Cronbach alpha (0.758) on a small pilot (n=15), which is acceptable but not robust. The cutoff for “adequate” proficiency (>6/12 marks) is based on the median score and expert opinion, which feels arbitrary and not clinically validated. Why not base it on critical steps alone, given their emphasis on efficacy and safety? Only 6% performed all 5 critical steps correctly, yet 47% were deemed “adequate” overall. This discrepancy suggests the threshold may be too lenient, masking true incompetence in high-impact areas like directing the nozzle away from the septum (to prevent epistaxis) or exhaling through the mouth (to optimize deposition). Please address these in the Discussion section.*

**Response:** Thank you for this comment. The 12-step intranasal corticosteroid checklist was developed from established international guidelines (eg, ARIA, Benninger et al [3], NHS), ensuring content relevance. As this tool assesses observed procedural technique, construct validity testing is not applicable. We have clarified this and acknowledged the limitation in the Discussion.

We agree the pilot sample was small. Cronbach alpha of 0.758 represents acceptable internal consistency for an observational checklist. The limitation has now been explicitly acknowledged.

The score of more than 6 is not arbitary; we conducted a sensitivity analysis using alternative cutoffs (>5 and >7). Receiver operating characteristic analysis could not be performed because the total score forms the derived outcome without an external gold standard. Sensitivity analysis showed that (1) predictors remained stable and significant at >5 and >6 and (2) the >7 cutoff produced unstable models due to sparse cell counts. Thus, the >6 threshold is empirically supported, aligns with >50% competency, the median distribution, and expert opinion. Relevant text was added to the Methods and Discussion.

Only 6% of participants completed all critical steps; using this as the cutoff would create extremely low event counts and make regression analysis unreliable. Moreover, international guidelines require all 12 steps for complete patient counseling. We expanded the Discussion to highlight the clinical significance of poor critical-step performance.

*3. Self-reported variables (eg, counseling frequency, use of materials) are prone to recall or social desirability bias, especially in an in-person interview setting. Please supplement these in the Discussion section.*

**Response:** Thank you for the concern. The risk of recall or social desirability bias is mentioned in the Discussion section.

*4. The multivariate binary logistic regression identifies associations (eg, male gender, older age, higher qualifications linked to better proficiency), but potential confounders like pharmacy type (independent vs chain) or workload details are not controlled for. Odds ratios are extreme in places (eg, BPharm holders 97% less likely to perform inadequately, or frequent counselors 11 times more proficient), which may stem from small subgroups or multicollinearity.*

**Response:** In Nepal, most of the pharmacies are independently owned and very few are chain pharmacies. In this study, only independent pharmacies were used, therefore pharmacy type is not one of the potential confounders in this study. However, workload details as potential confounders were not measured, which may have partly contributed to the large adjusted odds ratio of some predictors. It is mentioned in the Discussion.

*5. Gender differences (males ~2 times more proficient) were found but underlying factors were not explored (eg, access to workshops, cultural biases). Please elaborate more or address the potential underlying factors in the Discussion section.*

**Response:** We thank the reviewer for highlighting this point. Additional contextual explanation has been added to the Discussion to address potential underlying factors.

*6. “Educational materials” are linked to better proficiency, but what constitutes these (eg, leaflets, videos)? Please specify for readers to enhance the proficiency on applying the study’s results.*

**Response:** Thank you for the concern. The term “educational materials” mean the leaflet and now it is clearly mentioned in the Results.

*7. Reference 16 has the wrong format for the volume, issue, and page numbers:*

*Al-Taie A. A Systematic Review for Improper Application of Nasal Spray in Allergic Rhinitis: A Proposed Role of Community Pharmacist for Patient Education and Counseling in Practical Setting. Asia Pacific Allergy. 2025:10‐5415.*

*The full information from PubMed is as below:*

*Al-Taie A. A systematic review for improper application of nasal spray in allergic rhinitis: A proposed role of community pharmacist for patient education and counseling in practical setting. Asia Pac Allergy. 2025 Mar;15(1):29‐35. doi: 10.5415/apallergy.0000000000000173. Epub 2025 Jan 13. PMID: 40051424; PMCID: PMC11882221.*

*Therefore, “2025:10‐5415” should be “2025 Mar;15(1):29‐35.”*

*Please revise the whole reference list to see if any other typos exist.*

**Response:** Thank you for the concern. All the references have been revised.

### Reviewer AL [4]

#### General Comments

*This is an important and well-written study. My suggestions are listed below.*

#### Specific Comments

*There are some problems with language and with unnecessary capitalization of words.*

*Page 3: INCS sprays should be defined in full on first mention in the text.*

**Response:** Thank you for the concern. INCS spray is defined in full on first mention.

*Page 8: Can details of the ethical committee that provided the approval be provided? Was the informed consent obtained in writing?*

**Response:** The ethical committee details are now added in the manuscript. Yes, written informed consent was obtained from the participants.

*Scoring system: Should the crucial steps not be provided with greater marks compared to the other steps?*

**Response:** We thank the reviewer for this insightful suggestion. Although the five steps marked as “critical” have a greater clinical impact on efficacy and safety, we deliberately assigned equal weight (1 mark per step) to all 12 steps to maintain consistency with previously published studies that used similar checklist-based scoring systems and to avoid introducing subjective weighting without formal validation.

To address the clinical importance of critical steps, we analyzed them separately and reported their performance independently. Notably, although 47.1% of pharmacists met the overall adequacy threshold, only 6% correctly demonstrated all five critical steps, highlighting a substantial gap that would have been masked even if weighted scoring were used.

We agree that weighted scoring systems may better reflect clinical risk; however, such systems require prior validation. We have therefore added this point to the Limitations section and recommend weighted or competency-based scoring models in future studies.

*Page 17: Please explain the classification tree (Chi-square automatic interaction detection method) for the benefit of the readers.*

**Response:** We thank the reviewer for this helpful suggestion. We have now added a brief explanation of the classification and regression tree analysis using the Chi-square automatic interaction detector method in the Statistical Analysis and Results sections. The revised text explains the purpose of the method, the basis of variable splitting, and how the resulting tree should be interpreted. This addition is intended to improve clarity and accessibility for readers who may be unfamiliar with decision tree–based methods.

*Page 17: “This research is one of a kind, conducted in Nepal.” Can this sentence be modified?*

**Response:** Thank you for the insightful suggestion. The sentence has been modified.

*Page 19: Instead of continuing medical education (CME), continuing pharmacy education (CPE) may be a better term.*

**Response:** Thank you for the suggestion. Continuing pharmacy education (CPE) has been used instead of continuing medical education (CME) in the manuscript.

*Page 20: What educational aids are you referring to?*

**Response:** Educational aids means the leaflets and that has been clarified in the manuscript now.

*Are the educational leaflets available in the Nepali language?*

**Response:** The educational leaflet was available in the English language and the pharmacist used it for reference while counseling the patients.

*Page 20: “In our study, both the increasing age (> 26 y old) were significantly associated with improved INCS [intranasal corticosteroid] counseling proficiency.” This sentence mentions both but then highlights only one factor.*

**Response:** Thank you for the suggestion. It was a typing error in the manuscript and has been corrected.

*Was this study conducted only in Kathmandu city and not in Lalitpur or Bhaktapur?*

**Response:** This study was extensively conducted only in Kathmandu district.

*Page 21, Limitations section: Some of the findings may be extreme due to small subgroups or model overfitting. Can this be explained?*

**Response:** Thank you for the suggestion. This has been explained in the Limitations section.

*Different fonts are used in different locations, and this should be corrected.*

**Response:** Thank you for the comment. Font size has been corrected.
